# Association of Daily Eating Duration and Day-To-Day Variability in the Timing of Eating With Fatal Cancer Risk in Older Men

**DOI:** 10.3389/fnut.2022.889926

**Published:** 2022-05-10

**Authors:** Elisa M. S. Meth, Lieve T. van Egmond, Thiago C. Moulin, Jonathan Cedernaes, Fredrik Rosqvist, Christian Benedict

**Affiliations:** ^1^Department of Surgical Sciences, Uppsala University, Uppsala, Sweden; ^2^Department of Experimental Medical Science, Lund University, Lund, Sweden; ^3^Department of Medical Sciences, Uppsala University, Uppsala, Sweden; ^4^Department of Medical Cell Biology, Uppsala University, Uppsala, Sweden; ^5^Department of Public Health and Caring Sciences, Clinical Nutrition and Metabolism, Uppsala University, Uppsala, Sweden; ^6^Sleep Science Laboratory, Department of Pharmaceutical Biosciences, Molecular Neuropharmacology, Uppsala University, Uppsala, Sweden

**Keywords:** meal timing, eating duration, eating midpoint, day-to-day variability in the timing of eating, cancer, cohort, hazard ratio

## Abstract

Meal timing has significant effects on health. However, whether meal timing is associated with the risk of developing and dying of cancer is not well-researched in humans. In the present study, we used data from 941 community-dwelling men aged 71 years who participated in the Uppsala Longitudinal Study of Adult Men to examine the association of meal timing with cancer morbidity and fatal cancer. The following meal timing variables were derived from 7-day food diaries: (i) daily eating duration, i.e., the time between the first and last eating episode of an arbitrary day; (ii) the calorically weighted midpoint of the daily eating interval, a proxy of when the eating window typically occurs during an arbitrary day; and (iii) the day-to-day variability in the timing of eating. We also assessed the reported daily energy intake reliability using the Goldberg method. During a mean observational period of 13.4 years, 277 men (29.4%) were diagnosed with cancer. Furthermore, 191 men (20%) died from cancer during 14.7 years of follow-up. As shown by Cox regression adjusted for potential confounders (e.g., smoking status and daily energy intake), men with reliable dietary reports whose daily eating intervals were on average 13 h long had a 2.3-fold greater fatal cancer risk than men whose daily eating windows were on average about 11 h long. We also found that men with an average day-to-day variability in the timing of eating of 48 to 74 min had a 2- to 2.2-fold higher fatal cancer risk than those with the lowest average day-to-day variability in the timing of eating (i.e., 23 min). No clear associations were found in men with inadequate dietary reports, emphasizing the need to consider the reliability of dietary records in nutritional epidemiology. To fully unlock its potential, studies are needed to test whether recommendations to time-restrict the 24-h eating interval and reduce day-to-day variability in the timing of eating can meaningfully alter the risk of death due to cancer.

## Introduction

Dietary habits have been recognized as a modifiable risk factor for many types of cancer. For example, a meta-analysis including more than three million participants demonstrated that high adherence to the Mediterranean diet was related to a lower risk of all-cause cancer mortality (1). Furthermore, a separate study including about 800,000 participants revealed that those who best complied with a dietary approach to stop hypertension (characterized by, e.g., low intake of saturated fat) had a 19% lower relative risk of colorectal cancer (2). Finally, in a prospective study from Sweden following about 1,000 community-dwelling older men over a median period of 13 years, dietary habits characterized by low carbohydrate and high protein intake were associated with a ~50% reduced risk to be diagnosed with prostate cancer (3). However, the association between diet and prostate cancer in this Swedish study was only seen among men with accurate reports of energy intake (3). Food intake reports of poor validity can distort the relationships between nutrient intake and health (4).

In addition to the type of diet, the 24-h temporal eating pattern may affect cancer risk in humans. For example, in a study among 2,413 women with early-stage breast cancer, a habitual short nightly fast of <13 h was associated with a 36% higher risk for breast cancer recurrence (5). Furthermore, in a case-control study, habitual fasting for more than 11 h overnight was associated with 23% lower odds of prostate cancer (6). In addition, a prospective French study involving 41,389 day-working men and women found that regular late eating, defined as an eating episode after 21:30 h, was associated with an increased breast and prostate cancer risk (+48 and +120%, respectively) (7). Finally, a population-based case-control study in Spain observed that those who habitually fasted at least 2 h before their nighttime rest had a 20% lower risk for breast and prostate cancer (8).

The cancer incidence is greater in older adults (9). However, it has not been systematically studied among seniors whether meal timing characteristics affect the risk of developing and dying from cancer. Here, we examined in 70-year-old men if the daily eating interval, the timing of the daily eating interval, and the day-to-day variability in the timing of eating are associated with the risk of being diagnosed with and dying of cancer. Furthermore, since unreliable food logs represent a source of bias in nutritional epidemiology (10), we paid particular attention to those with reliable dietary reports.

## Subjects and Methods

### Setting and Participants

The present study is based on data from the Uppsala Longitudinal Study of Adult Men (ULSAM; http://www.pubcare.uu.se/ulsam). This prospective cohort study was initiated in 1970 with the primary aim to identify cardiovascular disease and diabetes risk factors in middle-aged men. All men born between 1920 and 1924 and living in the Uppsala municipality were invited to participate in the study; 82% (*n* = 2,322) agreed to participate. The men were reinvestigated repeatedly until December 2015 (see http://www.pubcare.uu.se/ulsam). Between August 1991 and May 1995, 53% of the initial sample (*n* = 1,221) participated in the age 70 investigation, of whom 93% filled out a 7-day dietary record (*n* = 1,138). Dietary habits were not recorded at any other age timepoint in the ULSAM cohort study. The age-70 investigation therefore served as the baseline. Following exclusions (e.g., a cancer diagnosis before baseline), 941 men remained for analysis. No imputation for missing values was performed (please see Supplementary Figure 1 for a summary of all exclusions). The regional ethics review board at Uppsala University approved the study, including all data reported herein (ethical diary numbers: 251/90 and 97/329). All participants gave informed consent before the study inclusion.

### Dietary Logs and Meal Timing Variables

After being instructed by a dietician, participants documented the content and time of each eating episode for one entire week. As previously suggested, we defined an eating episode as a calorie intake of at least 50 kcal (11). Based on the 7-day food logs, the three following meal timing variables of interest were computed:

i) The daily eating interval was calculated as the time between the first (after midnight) and last (before or at midnight) entry of an arbitrary day in the food diary (e.g., first entry at 08:00 and last entry at 18:00 = 10-h eating interval). The resulting seven daily eating intervals were then averaged.
ii) The midpoint of the daily eating interval was defined as half the time between the first and last eating episode of an arbitrary day and represents a proxy of when the eating window occurs during the 24-h day. The time of each eating episode was weighted for calorie content before calculating daily eating interval midpoints. For example, assume a person had breakfast at 06:00 (600 kcal), lunch at noon (600 kcal), and dinner at 18:00 (800 kcal) on an arbitrary day. In this case, the unweighted midpoint is at 12:00 ([06:00 + 12:00 + 18:00]/3), whereas the calorically weighted midpoint is at 12:36 (06:00^*^600 kcal/2,000 kcal + 12:00^*^600 kcal/2,000 kcal + 18:00^*^800 kcal/2,000 kcal). The one-week average for the calorically weighted midpoint was computed for the analysis.
iii) To determine the day-to-day variability in the timing of eating, we calculated the standard deviation of the seven daily calorically weighted midpoints. Thus, the lower the day-to-day variability in the timing of eating, the greater the eating midpoint regularity across the 7 days.

A mismatch between the timing of the eating window on working and free days, also called eating jetlag, has been associated with higher BMI (12). In the present study, we did not examine the association of eating jetlag with cancer outcomes, as the included men all passed the retirement age at the time of the investigation. Finally, as more than 90% of the participants had their last eating episode at 18:00, we did not examine the association of timing of the last meal with cancer outcomes.

Adults misreport their dietary intake on self-administered tools, most often in the direction of underreporting energy intake. Hence, we used the Goldberg approach modified by Black to identify men with inaccurate reports of energy intake (4). This method classifies participants as underreporters, accurate reporters, or overreporters using the ratio of reported energy intake to total energy expenditure. We calculated the total energy expenditure of each participant from the product of physical activity level (four categories as described below, physical activity factor: 1.4–1.7) and basal metabolic rate [using the age-adjusted Schofields formula, ref. (13)]. Then, a 95% confidence interval was created about the log of the ratio, and individuals who fell outside of the confidence interval were classified as participants inaccurately reporting daily energy intake.

### Cancer Outcomes

We used entries from the Swedish National Cancer and Swedish Cause of Death Registries to calculate the time at risk for primary cancer morbidity and fatal cancer (date of retrieval: December 31, 2013). The time at risk was calculated from the exact age at the baseline investigation (scheduled between August 1991 and May 1995) to the exact age of primary cancer diagnosis (for the primary cancer morbidity analysis), death due to cancer (for the fatal cancer analysis), actual age at last follow-up (December 31, 2013), or date of death due to something else than cancer, whichever came first.

### Confounders

Potential confounders measured during the age-70 investigation were selected based on previous literature (2, 14–18). Diabetes was treated as a binary variable and was confirmed when 2-h blood glucose readings during an oral glucose tolerance test were ≥11.1 mmol/l. The body mass index (BMI; kg/m^2^) was measured during the physical examination and treated as a continuous variable. Self-reported leisure-time physical activity was divided into four categories: mainly sedentary behavior (level 1); walking or cycling for pleasure (level 2), recreational sports or heavy gardening at least 3 h every week (level 4); and regularly engage in hard physical training (level 4). Participants' exact age (continuous), current smoking status (binary variable), and family history of cancer (any cancer diagnosis among parents or siblings) at the time of the baseline investigation were assessed via questionnaires or during an onsite interview. We used the 7-day food logs to estimate the Healthy Diet Indicator (HDI) score, which is based on the dietary guidelines from the World Health Organization (19). As described elsewhere (20), the HDI score ranged from −1 (minimum adherence level) to 8 points (maximum adherence level). Based on the 7-day food logs, each participant's daily energy intake was assessed to calculate the average across the 7 days of food registration. To determine participants' alcohol intake, for each day, the energy amount of alcohol was calculated and expressed as a percentage from the daily energy intake. For the analysis, the average across the 7 days was used (continuous variable). Finally, the season of the dietary recordings was assessed.

### Statistical Analysis

We performed all analyses with SPSS 26.0 (SPSS, Inc., Chicago, IL, USA). For the analysis, the meal timing variables were divided into quartiles (i.e., ≤25^th^ [quartile 1]; >25^th^ and ≤50^th^ [quartile 2]; >50^th^ and ≤75^th^ [quartile 3]; and >75^th^ percentile [quartile 4]; see Table 1 for descriptive statistics). To account for the reliability of the meal logs, we calculated quartiles separately for the subgroup with accurate and the subgroup with inaccurate reports of daily energy intake.

**Table 1:** Characteristics of the ULSAM cohort at the age 70 investigation.

**Parameter**	**Total cohort**	**Reliability of the energy intake report according to the Goldberg method** **(4)**	
		**Good**	**Poor**
Number of subjects	941	496	445
Age, years, mean ± SD	71.0 ± 0.6	71.0 ± 0.6	71.0 ± 0.6
Body mass index, kg/m^2^, mean ± SD	26.1 ± 3.2	25.1 ± 2.9	27.2 ± 3.1 ^**^
Healthy Diet Indicator score (min −1, max +8), mean ± SD	3.61 (1.20)	3.61 (1.11)	3.60 (1.29)
History of diabetes, %	13.9	12.1	16
Current smoker, %	19.9	18.8	21.1
Family history of cancer, %	40.4	40.1	40.7
**Physical activity status (the higher, the more physically active)**			
Level 1, %	3.3	3.8	2.7
Level 2, %	34.8	35.5	33.9
Level 3, %	55.6	56.0	55.1
Level 4, %	6.4	4.6	8.3
**Length of the daily eating interval**			
Entire group, hh:mm, mean ± SD	10:56 ± 01:27	11:11 ± 01:23	10:40 ± 01:28^**^
Quartile 1 (≤25^th^ percentile), hh:mm, mean ± SD	09:11 ± 00:47	09:27 ± 00:42	08:53 ± 00:45
Quartile 2 (>25^th^ and ≤50^th^ percentile), hh:mm, mean ± SD	10:28 ± 00:23	10:44 ± 00:17	10:11 ± 00:14
Quartile 3 (>50^th^ and ≤75^th^ percentile), hh:mm, mean ± SD	11:31 ± 00:29	11:50 ± 00:23	11:10 ± 00:20
Quartile 4 (>75^th^ percentile), hh:mm, mean ± SD	12:51 ± 00:30	13:01 ± 00:21	12:40 ± 00:34
**Calorically weighted midpoint of the daily eating interval**			
Entire group, hh:mm, mean ± SD	12:57 ± 00:45	13:00 ± 00:43	12:54 ± 00:48 ^*^
Quartile 1 (≤25^th^ percentile), hh:mm, mean ± SD	12:01 ± 00:26	12:09 ± 00:22	11:52 ± 00:27
Quartile 2 (>25^th^ and ≤50^th^ percentile), hh:mm, mean ± SD	12:43 ± 00:08	12:46 ± 00:07	12:40 ± 00:09
Quartile 3 (>50^th^ and ≤75^th^ percentile), hh:mm, mean ± SD	13:10 ± 00:09	13:10 ± 00:09	13:09 ± 00:09
Quartile 4 (>75^th^ percentile), hh:mm, mean ± SD	13:55 ± 00:25	13:57 ± 00:23	13:53 ± 00:27
**Day-to-day variability in the timing of eating**			
Entire group, hh:mm, mean ± SD	00:47 ± 00:23	00:45 ± 00:21	00:51 ± 00:24^**^
Quartile 1 (≤25^th^ percentile), hh:mm, mean ± SD	00:24 ± 00:06	00:23 ± 00:05	00:25 ± 00:06
Quartile 2 (>25^th^ and ≤50^th^ percentile), hh:mm, mean ± SD	00:37 ± 00:05	00:34 ± 00:03	00:40 ± 00:03
Quartile 3 (>50^th^ and ≤75^th^ percentile), hh:mm, mean ± SD	00:51 ± 00:06	00:48 ± 00:05	00:53 ± 00:05
Quartile 4 (>75^th^ percentile), hh:mm, mean ± SD	01:18 ± 00:20	01:14 ± 00:18	01:23 ± 00:20
Daily energy intake, kcal, mean ± SD	1,745 ± 457	2,047 ± 346	1,408 ± 307^**^
Alcohol intake, % of daily energy intake, mean ± SD	2.7 ± 3.2	2.3 ± 2.7	3.2 ± 2.7
Season at dietary recording, *n* (%)			
Spring	326 (34.6)	166 (33.5)	160 (36.0)
Summer	119 (12.6)	60 (12.1)	59 (13.3)
Autumn	184 (19.6)	101 (20.4)	83 (18.7)
Winter	312 (33.2)	169 (34.1)	143 (32.1)

*Possible group differences were analyzed either with univariate generalized linear models or chi-square-tests*.

* *P < 0.05 and*

** *P < 0.001 for accurate vs. inaccurate reports of energy intake*.

*SD, standard deviation; hh, hours; mm, minutes. To convert kcal to kJ, multiply kcal with 4.184*.

The association between the meal timing variables and cancer was examined by using Cox proportional hazard regression models with attained age (in days) as the underlying timescale to estimate hazard ratios (HRs) with 95% confidence intervals (CIs). Outcomes besides that being investigated in the analysis at present were handled as censoring event (for the primary cancer diagnosis, deaths were censored and for the fatal cancer analysis, deaths due to something else than cancer were censored). The crude Cox model included the daily eating interval, the calorically weighted eating midpoint, and the day-to-day variability in the timing of eating as independent variables. To test the robustness of potential associations with cancer outcomes, we added participants' age, BMI, family history of cancer status, smoking status, self-reported physical activity level, alcohol consumption, diabetes status, adherence to the healthy diet recommendations by the WHO, mean energy intake, and season of the dietary records to the fully adjusted Cox regression model. Since unreliable food logs represent a source of bias in nutritional epidemiology (10), we report results from the Cox regression analyses for men with and without reliable dietary reports separately. Proportional hazards assumptions were confirmed using graphical evaluation.

## Results

### Cohort Characteristics

**Figure 1:**
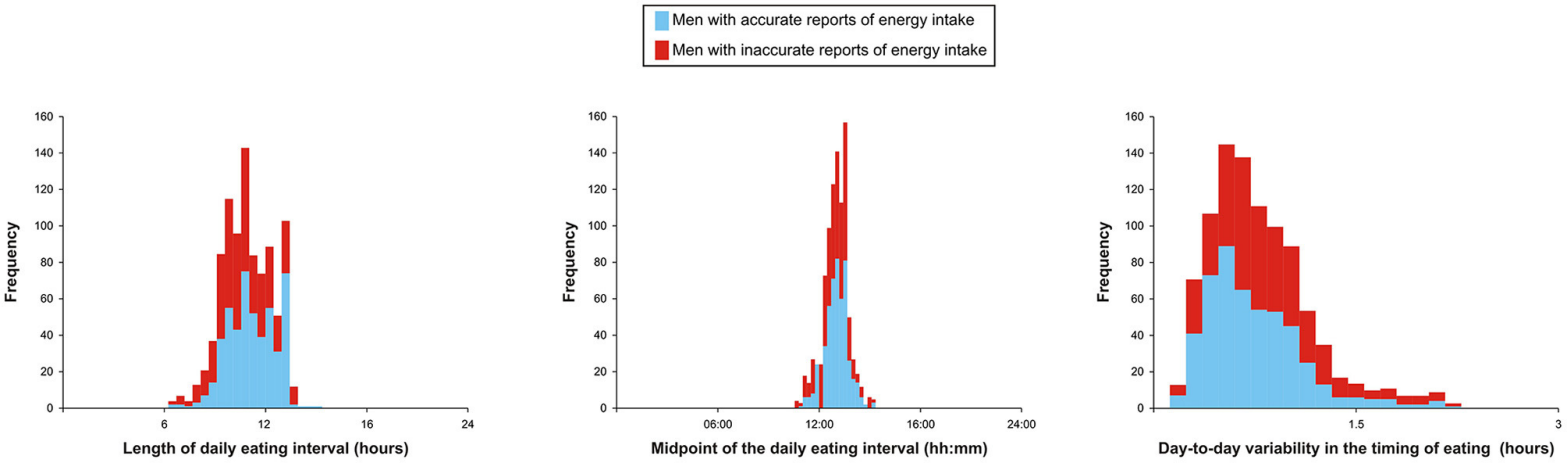
Histograms for meal timing variables split by reliability of the dietary records.

At baseline, the cohort was on average 71 years old, 10.8% of the men had a BMI ≥30kg/m^2^, and every fifth participant smoked. More characteristics, including a visualization of the distribution of the three meal timing variables of interest, can be found in Table 1 and Figure 1.

### Meal Timing Characteristics and Cancer Risk

During the mean observational period of 13.4 ± 5.8 years, 277 men (29.4% of the entire cohort) developed cancer (a list of primary cancer sites can be found in Supplementary Table 1). In addition, about 20% (*n* = 191) of the cohort died from cancer during the 14.7 ± 5.2 years follow-up (a list of cancer sites leading to death is shown in Supplementary Table 2).

Among men with reliable dietary reports, the fully adjusted Cox regression analysis revealed that men in the fourth quartile of the daily eating interval (meaning that their daily eating interval was about 13 h long) had a 2.33-fold higher risk of dying from cancer than those in the lowest quartile (i.e., those eating on average 11 h and 11 min; Table 2). We also found that men with an average a day-to-day variability in the timing of eating of 48–74 min (i.e., those assigned to quartiles 3 and 4) exhibited a 1.95- to 2.19-fold higher fatal cancer risk than those with the lowest the day-to-day variability in the timing of eating (i.e., those assigned to quartile 1; Table 2). In contrast, no significant associations between the quartiles of the meal timing exposures and cancer morbidity were observed (Table 3).

**Table 2:** Association of meal timing variables with cancer mortality among men with and without reliable dietary reports.

	**Number**	**Crude model** ^§^				**Adjusted model** ^†^			
		**Quartile 1**	**Quartile 2**	**Quartile 3**	**Quartile 4**	**Quartile 1**	**Quartile 2**	**Quartile 3**	**Quartile 4**
**Men with reliable dietary reports**	496	–	–	–	–	–	–	–	–
Fatal cancer events	98	–	–	–	–	–	–	–	–
Days at risk	2,741,996	–	–	–	–	–	–	–	–
Length of daily eating interval	–	1 (Ref)	1.56 [0.84, 2.90]	1.15 [0.60, 2.22]	**2.22 [1.18, 4.18]**	1 (Ref)	1.75 [0.94, 3.30]	1.40 [0.72, 2.72]	**2.33 [1.22, 4.44]**
Midpoint of daily eating interval	–	1 (Ref)	0.90 [0.52, 1.56]	0.75 [0.42, 1.33]	1.08 [0.59, 1.98]	1 (Ref)	0.90 [0.51, 1.59]	0.81 [0.45, 1.44]	1.16 [0.62, 2.17]
Day-to-day variability in the timing of eating	–	1 (Ref)	1.73 [0.95, 3.14]	1.46 [0.79, 2.69]	1.70 [0.93, 3.13]	1 (Ref)	1.75 [0.95, 3.25]	**1.95 [1.03, 3.68]**	**2.19 [1.16, 4.15]**
**Men with unreliable dietary reports**	445	–	–	–	–	–	–	–	–
Fatal cancer events	93	–	–	–	–	–	–	–	–
Days at risk	2,307,123	–	–	–	–	–	–	–	–
Length of daily eating interval	–	1 (Ref)	0.92 [0.53, 1.62]	0.63 [0.35, 1.12]	0.84 [0.47, 1.48]	1 (Ref)	0.81 [0.46, 1.45]	**0.51 [0.28, 0.95]**	0.79 [0.43, 1.44]
Midpoint of daily eating interval	–	1 (Ref)	1.12 [0.64, 1.98]	0.99 [0.55, 1.78]	0.93 [0.51, 1.70]	1 (Ref)	1.25 [0.70, 2.25]	1.00 [0.55, 1.83]	0.88 [0.47, 1.65]
Day-to-day variability in the timing of eating	–	1 (Ref)	1.13 [0.64, 2.01]	1.02 [0.57, 1.83]	1.14 [0.64, 2.02]	1 (Ref)	1.18 [0.65, 2.16]	1.01 [0.55, 1.85]	0.98 [0.53, 1.81]

§ *Including the length of the daily eating interval, the calorically weighted midpoint of the daily eating interval, and the day-to-day variability in the timing of eating*.

† *Adjusted for age, BMI, family history of cancer, smoking status, physical activity level, alcohol consumption, diabetes, healthy diet indicator score, season at dietary recording, and mean energy intake*.

*CI, confidence interval. Significant values are shown in bold*.

**Table 3:** Association of meal timing variables with primary cancer morbidity among men with and without reliable dietary reports.

	**Number**	**Crude model** ^§^				**Adjusted model** ^†^			
		**Quartile 1**	**Quartile 2**	**Quartile 3**	**Quartile 4**	**Quartile 1**	**Quartile 2**	**Quartile 3**	**Quartile 4**
**Men with reliable dietary reports**	496	–	–	–	–	–	–	–	–
Primary cancer events	146	–	–	–	–	–	–	–	–
Days at risk	2,495,237	–	–	–	–	–	–	–	–
Length of daily eating interval	–	1 (Ref)	1.21 [0.76, 1.94]	0.80 [0.48, 1.34]	1.46 [0.90, 2.38]	1 (Ref)	1.24 [0.77, 2.01]	0.78 [0.46, 1.31]	1.38 [0.84, 2.30]
Midpoint of daily eating interval	–	1 (Ref)	0.97 [0.62, 1.51]	0.63 [0.39, 1.02]	1.03 [0.64, 1.66]	1 (Ref)	0.98 [0.62, 1.55]	0.63 [0.38, 1.03]	0.96 [0.57, 1.59]
Day-to-day variability in the timing of eating	–	1 (Ref)	1.16 [0.73, 1.84]	1.19 [0.76, 1.88]	0.98 [0.60, 1.60]	1 (Ref)	1.21 [0.75, 1.95]	1.29 [0.81, 2.07]	1.10 [0.66, 1.83]
**Men with unreliable dietary reports**	445	–	–	–	–	–	–	–	–
Primary cancer events	131	–	–	–	–	–	–	–	–
Days at risk	2,114,099	–	–	–	–	–	–	–	–
Length of daily eating interval	–	1 (Ref)	0.93 [0.57, 1.52]	0.73 [0.45, 1.19]	0.98 [0.61, 1.59]	1 (Ref)	0.91 [0.56, 1.50]	0.74 [0.45, 1.21]	1.05 [0.63, 1.74]
Midpoint of daily eating interval	–	1 (Ref)	1.54 [0.97, 2.45]	0.75 [0.44, 1.28]	1.06 [0.64, 1.75]	1 (Ref)	**1.69 [1.05, 2.71]**	0.78 [0.46, 1.35]	1.22 [0.73, 2.05]
Day-to-day variability in the timing of eating	–	1 (Ref)	0.97 [0.59, 1.59]	0.96 [0.59, 1.54]	0.99 [0.61, 1.60]	1 (Ref)	0.97 [0.58, 1.62]	0.94 [0.57, 1.54]	0.86 [0.51, 1.43]

§ *Including the length of the daily eating interval, the calorically weighted midpoint of the daily eating interval, and the day-to-day variability in the timing of eating*.

† *Adjusted for age, BMI, family history of cancer, smoking status, physical activity level, alcohol consumption, diabetes, healthy diet indicator score, season at dietary recording, and mean energy intake*.

*CI, confidence interval. Significant values are shown in bold*.

The associations of the meal timing parameters with cancer outcomes among men with unreliable dietary reports are summarized in Tables 2, 3.

## Discussion

Several studies have shown that short regular daily eating intervals (definitions range between ≤11 and <13 h) reduce the risk of cancer and prolong the cancer remission period among cancer survivors (5, 6). However, whether meal timing characteristics, such as the length and timing of the daily eating interval and regularity of daily eating patterns, are associated with the risk of developing and dying from cancer in men of advanced age is largely unknown. In the present prospective cohort of community-dwelling older men with a maximum follow-up of over 22 years, we show that those eating on average 13 h per day and exhibiting an average day-to-day variability in the timing of eating of about 50 min or more have an increased risk for fatal cancer. These associations were seen despite adjustment for well-known cancer risk factors, e.g., smoking, dietary habits, and daily energy intake. If confirmed in future studies with larger sample size that involve assessment at a younger age and both sexes, our results suggest that restricting the daily eating interval and reducing the day-to-day variability in the timing of eating may have some potential in the prevention of fatal cancer among seniors.

The observation that the timing of the daily eating interval, measured by its midpoint, was not associated with cancer contrasts with some but not all previous studies. For example, a shorter daytime eating window has been shown to reduce the risk of prostate cancer in those starting eating at 08:30 or before. However, no such association was seen for those having a later onset of the daily eating interval (6), suggesting that not only the length but also the timing of the eating interval may impact the future cancer risk. However, a study among breast cancer survivors showed that fasting at least 13 h per day reduced the risk of breast cancer recurrence, irrespective of the timing of the offset of the eating interval (5).

Although our study cannot establish causality, several possible mechanisms could explain the association of the length of the daily eating duration and the day-to-day variability in the timing of eating with the risk of fatal cancer. For example, even during a shorter fasting period that can occur within a single day, healthy cells can switch from an anabolic state toward maintenance and repair state. This may limit the growth potential and mutagenic process of pre-malignant or cancerous cells (21). In contrast, proliferating cancer cells fail to adapt to scarce nutrient conditions because of a relative increase in their metabolic activity (22). Furthermore, fasting decreases blood levels of insulin-growth factor I (23, 24), a hormone that activates cellular oncogenes involved in malignant cell proliferation (25, 26). These anticancer properties may also explain why intermittent fasting has attracted significant interest as a potential lifestyle strategy in the prevention and treatment of cancer (27, 28). However, it must be kept in mind that results from intermittent fasting studies in rodents are controversial and suggest potential detrimental effects in certain oncological conditions (29). Finally, a greater day-to-day variability in the timing of eating may concur with a greater risk that food intake coincides with metabolically inappropriate endogenous circadian times. Notably, a temporal mismatch between behavioral and endogenous metabolic rhythms, e.g., as occurs in jetlag, has been associated with cancer in animal models (30).

Several strengths apply to our study, including the prospective study design and the extended follow-up, and that the observed associations remained significant following adjustment for well-known cancer risk factors. However, limitations of our study include the observational design and that residual confounding cannot be excluded. Furthermore, no potential site-specific associations between cancer outcomes and meal timing characteristics were examined. Additionally, dietary habits were only monitored at the age-70 investigation. The present study involved only men. Thus, it remains unclear whether meal timing characteristics affect cancer outcomes in women, although preliminary evidence is in favor of this hypothesis. For example, a daily eating interval shorter than 13 h may prolong cancer remission, as suggested by an investigation of female breast cancer patients (5). Finally, 29% of the cohort developed cancer during the observational period, and 20% died with cancer as the primary cause. These numbers are higher than those usually reported by, e.g., the U.S. National Cancer Institute (9), and could be explained by selection bias. With all these limitations in mind, other population-based studies, including women and repeated assessments of dietary characteristics, are needed to evaluate the generalizability of our findings further.

According to the widely applied Goldberg cut-off criterion (4), ~47% of the participants in the present study provided inaccurate reports of energy intake. Compared to men with accurate reports of daily energy intake, those with unreliable food logs had a two-point greater BMI and an about 31% lower daily energy intake. The magnitude of misreporting of energy intake by older men in the present study was greater than typically seen for younger men. For example, the percentage of underreporters in studies using estimated food records ranged from 14.3 to 42% for men (31). The underlying reasons for the relatively high proportion of older men with inaccurate energy intake reports in the present study are unclear. Still, they may include respondent memory lapses and misrepresentation of portion size consumed. Thus, screening for unreliable dietary reports is necessary when studying the association of nutritional patterns, including meal timing, with health outcomes, especially when including seniors. If ignored, inaccurate dietary reports can otherwise contribute to the lack of replicability and inconsistency across studies in the field of nutritional research, including nutritional epidemiology (10, 32). Future studies should also compare the efficacy of various methods to measure food intake among seniors reliably.

## Conclusions

Our study indicates that curtailing the length of the daily eating interval and reducing the day-to-day variability in the timing of eating may be potential behavioral strategies to reduce the fatal cancer risk among older men. However, to fully unlock its potential, studies are needed to test whether recommendations to time-restrict the 24-h eating interval and have regularly timed daily eating intervals can reduce the risk of death due to cancer.

## Data Availability Statement

The original contributions presented in the study are included in the article/Supplementary Materials, further inquiries can be directed to the corresponding author/s.

## Ethics Statement

The studies involving human participants were reviewed and approved by the Regional Ethics Review Board at Uppsala University (https://etikprovningsmyndigheten.se/). The patients/participants provided their written informed consent to participate in this study.

## Author Contributions

EM and CB came up with the research idea. EM, LvE, TM, and CB analyzed data. EM wrote the paper. CB had primary responsibility for final content. All authors read and approved the final manuscript.

## Funding

The authors' work is funded by the Novo Nordisk Foundation (NNF19OC0056777) and Swedish Brain Research Foundation (FO2020-0044). The funding organizations had no role in the design and conduct of the study; collection, management, analysis, and interpretation of the data; preparation, review, or approval of the manuscript; and decision to submit the manuscript for publication.

## Conflict of Interest

The authors declare that the research was conducted in the absence of any commercial or financial relationships that could be construed as a potential conflict of interest.

## Publisher's Note

All claims expressed in this article are solely those of the authors and do not necessarily represent those of their affiliated organizations, or those of the publisher, the editors and the reviewers. Any product that may be evaluated in this article, or claim that may be made by its manufacturer, is not guaranteed or endorsed by the publisher.
