# Supplementary material for: Association of Daily Eating Duration and Day-To-Day Variability in the Timing of Eating With Fatal Cancer Risk in Older Men
Source: Front Nutr. 2022 May 10;9:889926. doi: 10.3389/fnut.2022.889926 (PMC9127957; doi:10.3389/fnut.2022.889926)
Supplement: Supplementary file 1 [file Data_Sheet_1.docx]

**Figure S1. Flowchart of exclusions**

**
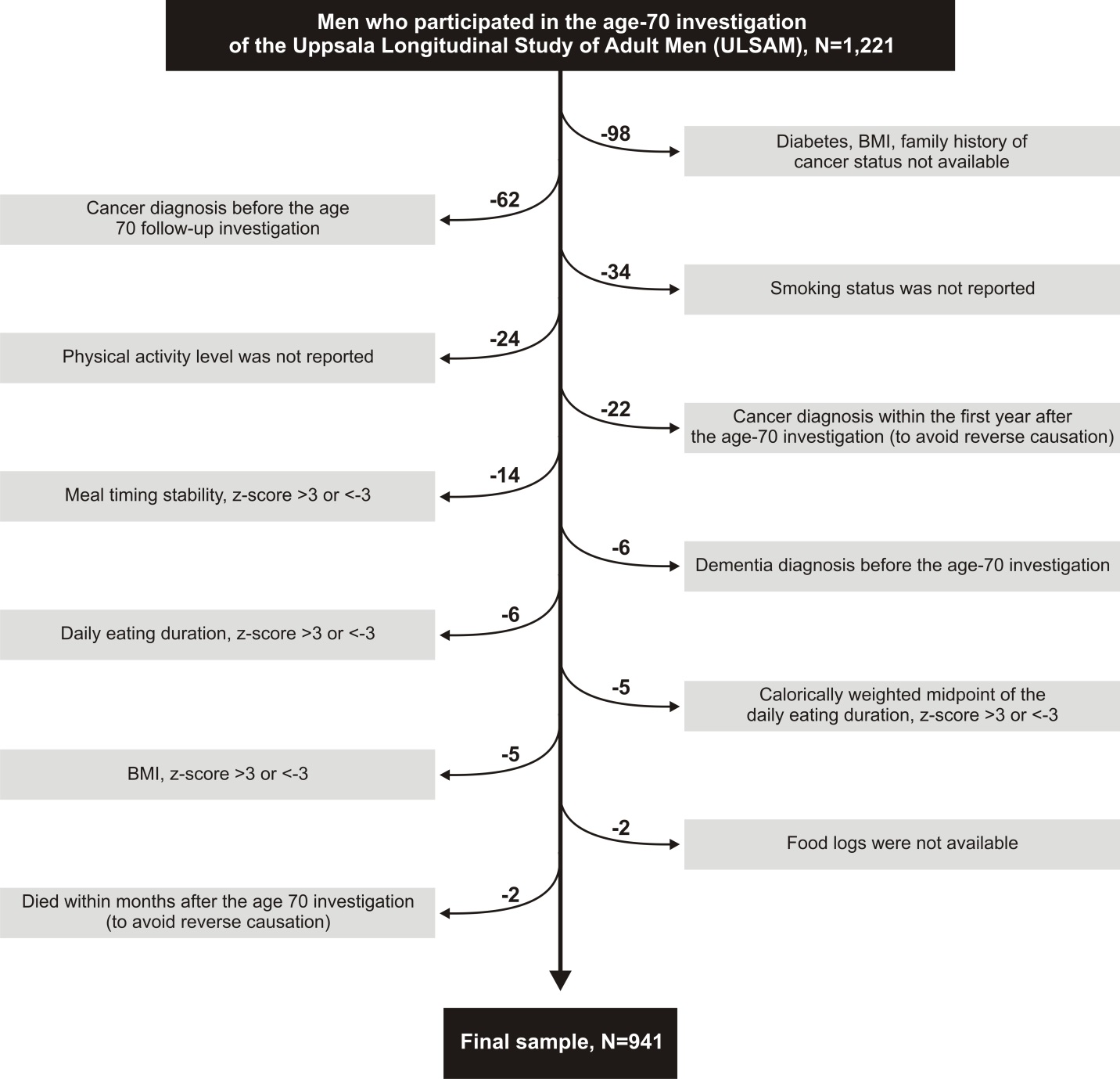
**

| **Supplemental Table S1: Distribution of incident cancer cases by site** | | |
| --- | --- | --- |
| **Primary cancer site** | **ICD-7** | **Cases during observational period** |
| Malignant neoplasm of lip | 140 | 2 |
| Malignant neoplasm of other parts of mouth, and of mouth, unspecified | 144 | 1 |
| Malignant neoplasm of hypopharynx | 147 | 1 |
| Malignant neoplasm of oesophagus | 150 | 1 |
| Malignant neoplasm of stomach | 151 | 11 |
| Malignant neoplasm of small intestine, including duodenum | 152 | 1 |
| Malignant neoplasm of large intestine, except rectum | 153 | 23 |
| Malignant neoplasm of rectum | 154 | 10 |
| Malignant neoplasm of biliary passages and of liver (stated to be primary site) | 155 | 2 |
| Malignant neoplasm of pancreas | 157 | 6 |
| Malignant neoplasm of nose, nasal cavities, middle ear and accessory sinuses | 160 | 1 |
| Malignant neoplasm of larynx | 161 | 1 |
| Malignant neoplasm of bronchus and trachea, and of lung specified as primary | 162 | 25 |
| Malignant neoplasm of prostate | 177 | 111 |
| Malignant neoplasm of kidney | 180 | 3 |
| Malignant neoplasm of bladder and other urinary organs | 181 | 26 |
| Malignant melanoma of skin | 190 | 8 |
| Malignant neoplasm of eye | 192 | 1 |
| Malignant neoplasm of brain and other parts of nervous system | 193 | 1 |
| Malignant neoplasm of thyroid gland | 194 | 1 |
| Malignant neoplasm of bone (including jaw bone) | 195 | 1 |
| Malignant neoplasm of connective tissue | 197 | 2 |
| Malignant neoplasm of other and unspecified sites | 199 | 12 |
| Lymphosarcoma and reticulosarcoma | 200 | 10 |
| Other forms of lymphoma (reticulosis) | 202 | 1 |
| Multiple myeloma (plasmocytoma) | 203 | 6 |
| Leukaemia and aleukaemia | 204 | 6 |
| Mycosis fungoides | 205 | 2 |
| Myelofibrosis | 208 | 1 |
| *Abbreviations*: ICD-7, International Classification of Diseases, Revision 7 | | |

| **Supplemental Table S2: Deaths caused by cancer** | | |
| --- | --- | --- |
| **Primary cancer site** | **ICD-9/10** | **Cases during observational period** |
| Malignant neoplasm of stomach, unspecified site | 1519 | 1 |
| Malignant neoplasm of intestinal tract, part unspecified | 1590 | 1 |
| Malignant neoplasm of pleura, unspecified | 1639 | 1 |
| Malignant neoplasm of prostate | 185 | 1 |
| Malignant neoplasm of bladder, part unspecified | 1889 | 1 |
| Neoplasm of unspecified nature of brain | 2396 | 1 |
| Malignant neoplasm of esophagus, unspecified | C15.9 | 4 |
| Malignant neoplasm of cardia | C16.0 | 2 |
| Malignant neoplasm of stomach, unspecified | C16.9 | 10 |
| Malignant neoplasm of duodenum | C17.0 | 1 |
| Malignant neoplasm of cecum | C18.0 | 1 |
| Malignant neoplasm of ascending colon | C18.2 | 1 |
| Malignant neoplasm of colon, unspecified | C18.9 | 10 |
| Malignant neoplasm of rectosigmoid junction | C19.0 | 1 |
| Malignant neoplasm of rectum | C20.0 | 3 |
| Malignant neoplasm: Liver, unspecified | C22.9 | 1 |
| Malignant neoplasm: Extrahepatic bile duct | C24.0 | 1 |
| Malignant neoplasm of head of pancreas | C25.0 | 2 |
| Malignant neoplasm of pancreas, unspecified | C25.9 | 11 |
| Malignant neoplasm of intestinal tract, part unspecified | C26.9 | 1 |
| Malignant neoplasm of upper lobe, bronchus or lung | C34.1 | 1 |
| Malignant neoplasm of unspecified part of bronchus or lung | C34.9 | 23 |
| Malignant melanoma of skin, unspecified | C43.9 | 2 |
| Malignant neoplasm of connective and soft tissue of lower limb, including hip | C49.2 | 1 |
| Malignant neoplasm of prostate | C61.0 | 60 |
| Malignant neoplasm of kidney, except renal pelvis | C64.0 | 2 |
| Malignant neoplasm of renal pelvis | C65.0 | 1 |
| Malignant neoplasm of bladder, unspecified | C67.9 | 12 |
| Malignant neoplasm of brain, unspecified | C71.9 | 2 |
| Malignant neoplasm of thyroid gland | C73.0 | 1 |
| Malignant neoplasm of abdomen | C76.2 | 1 |
| Disseminated malignant neoplasm, unspecified | C80.0 | 4 |
| Malignant neoplasm, primary site unspecified. | C80.9 | 2 |
| Diffuse large B-cell lymphoma | C83.3 | 1 |
| Other non-follicular lymphoma | C83.8 | 1 |
| Mature T/NK-cell lymphomas | C84.05 | 2 |
| Other specified and unspecified types of non-Hodgkin lymphoma | C85.1 | 1 |
| Other specified and unspecified types of non-Hodgkin lymphoma | C85.9 | 2 |
| Chronic lymphocytic leukemia of B-cell type | C91.1 | 2 |
| Acute myeloblastic leukemia | C92.0 | 1 |
| Leukemia, unspecified | C95.9 | 2 |
| Malignant neoplasms of independent (primary) multiple sites | C97 | 2 |
| Neoplasm of uncertain behavior of stomach | D37.1 | 1 |
| Neoplasm of uncertain behavior of colon | D37.4 | 1 |
| Neoplasm of uncertain or unknown behaviour: Liver, gallbladder and bile ducts | D37.6 | 2 |
| Neoplasm of uncertain/unknown behaviour: other digestive organs | D37.7 | 1 |
| Neoplasm of uncertain behavior of renal pelvis | D41.1 | 1 |
| Neoplasm of uncertain behavior of brain | D43.2 | 2 |
| Myelodysplastic syndrome | D46.9 | 1 |
| Neoplasm of uncertain behavior of other specified sites | D48.7 | 1 |
| Abbreviations: ICD-9/10, International Classification of Diseases, Revision 9/10 | | |
